# Cost-Consequence Analysis Alongside a Randomised Controlled Trial of Hospital Versus Telephone Follow-Up after Treatment for Endometrial Cancer

**DOI:** 10.1007/s40258-018-0378-6

**Published:** 2018-04-12

**Authors:** Padraig Dixon, Kinta Beaver, Susan Williamson, Chris Sutton, Pierre Martin-Hirsch, William Hollingworth

**Affiliations:** 1 Population Health Sciences, Bristol Medical School, University of Bristol, Canynge Hall, 39 Whatley Road, Bristol, BS8 2PS UK; 2 MRC Integrative Epidemiology Unit, University of Bristol, Oakfield House, Oakfield Grove, Bristol, BS8 2BN UK; 3 Faculty of Health and Wellbeing, University of Central Lancashire, Preston, PR1 2HE UK; 4 Department of Obstetrics and Gynaecology, Lancashire Teaching Hospitals, Preston, PR2 9HT UK

## Abstract

**Background:**

Regular outpatient follow-up programmes are usually offered to patients following treatment for gynaecological and other cancers. Despite the substantial resources involved in providing these programmes, there is evidence that routine follow-up programmes do not affect survival or the likelihood of detecting recurrence and may not meet patient needs. Alternative follow-up modalities may offer the same outcomes at lower cost. We examined the costs of using telephone-based routine follow-up of women treated for endometrial cancer undertaken by specialist gynaecology oncology nurses in comparison to routine hospital-based follow-up.

**Methods:**

The ENDCAT trial randomised 259 women at five centres in the north west of England with a known diagnosis of Stage I endometrial cancer who had completed primary treatment on a 1:1 basis to receive either standard hospital outpatient follow-up or a telephone follow-up intervention administered by specialist nurses. A cost-consequence analysis was undertaken in which we compared costs to the health system and to individuals with the trial’s co-primary outcomes of psychological morbidity and participant satisfaction with information received.

**Results:**

Psychological morbidity, psychosocial needs, patient satisfaction and quality of life did not differ between arms. Patients randomised to telephone follow-up underwent more and longer consultations. There was no difference in total health service mean per patient costs at 6 months (mean difference £8, 95% percentile confidence interval: − £147 to £141) or 12 months (mean difference: − £77, 95% percentile confidence interval: − £334 to £154). Estimated return journey costs per patient for hospital consultations were £11.47. Productivity costs were approximately twice as high under hospital follow-up.

**Conclusion:**

Telephone follow-up was estimated to be cost-neutral for the NHS and may free up clinic time for other patients. There was some evidence that telephone follow-up may be more efficient for patients and wider society, and is not associated with additional psychological morbidity, lower patient satisfaction or reduced quality of life.

**Trial Registration:**

ISRCTN: 75220876, prospectively registered 28 October 2011.

**Electronic supplementary material:**

The online version of this article (10.1007/s40258-018-0378-6) contains supplementary material, which is available to authorized users.

## Key Points for Decision Makers

**Table:** 

Telephone-based follow-up patients following treatment for gynaecological and other cancers is an acceptable alternative to regular hospital-based outpatient follow-up programmes.
The ENDCAT trial compared NHS and personal costs in up to 259 patients randomised to receive either hospital or telephone-based follow-up after treatment for Stage I endometrial cancer.
Telephone-based follow-up was no worse for psychological morbidity than hospital-based follow-up, was not more expensive to the health system than hospital-based follow-up and may be associated with lower personal costs.

## Background

Over two million people are currently living with and beyond cancer in the United Kingdom (UK), a figure estimated to rise to four million by 2030 [1]. Cancer-care pathways indicate that the highest numbers of patients are in the post-treatment rehabilitation and monitoring phases [2]. This follow-up stage usually involves hospital outpatient appointments on a regular basis for a number of years. The purpose of follow-up is to detect recurrence of cancer at an early stage with the aim of extending survival and/or improving quality of life [3]. Providing follow-up care post-treatment is likely to be extremely challenging, given the increasing numbers combined with finite healthcare resources. In addition, there is little evidence to support a survival advantage from hospital-based follow-up for different types of cancer [3–5]. A recent Cancer Taskforce report emphasised the need for efficiency savings and the evaluation of alternative approaches to hospital-based follow-up after treatment for cancer [6].

Alternative modalities of follow-up may offer a means of achieving better outcomes at lower cost [7]. This study describes an investigation of the costs and impact on psychological morbidity, patient satisfaction and quality of life of nurse-led, telephone-based follow-up in comparison to routine, doctor- or nurse-led, hospital-based outpatient appointments following treatment for Stage I endometrial cancer. Globally, there were estimated to be 320,000 incident cases of endometrial cancer in 2012, associated with 76,000 deaths [8]. Stage I endometrial cancer, which is confined to the uterus, accounts for 75% of all diagnoses of the disease [9], and is associated with mean 5-year survival of greater than 70% [10, 11]. Early-stage endometrial cancer has a low risk of recurrence and the majority of recurrences are symptomatic [12]. Hence, less clinically intensive follow-up approaches may be justified for this patient group.

The aim of the analysis was to estimate the costs and consequences of using telephone-based routine follow-up of women treated for endometrial cancer undertaken by specialist gynaecology oncology nurses in comparison to routine management using doctor-led hospital-based follow-up.

## Methods

**Fig. 1:**
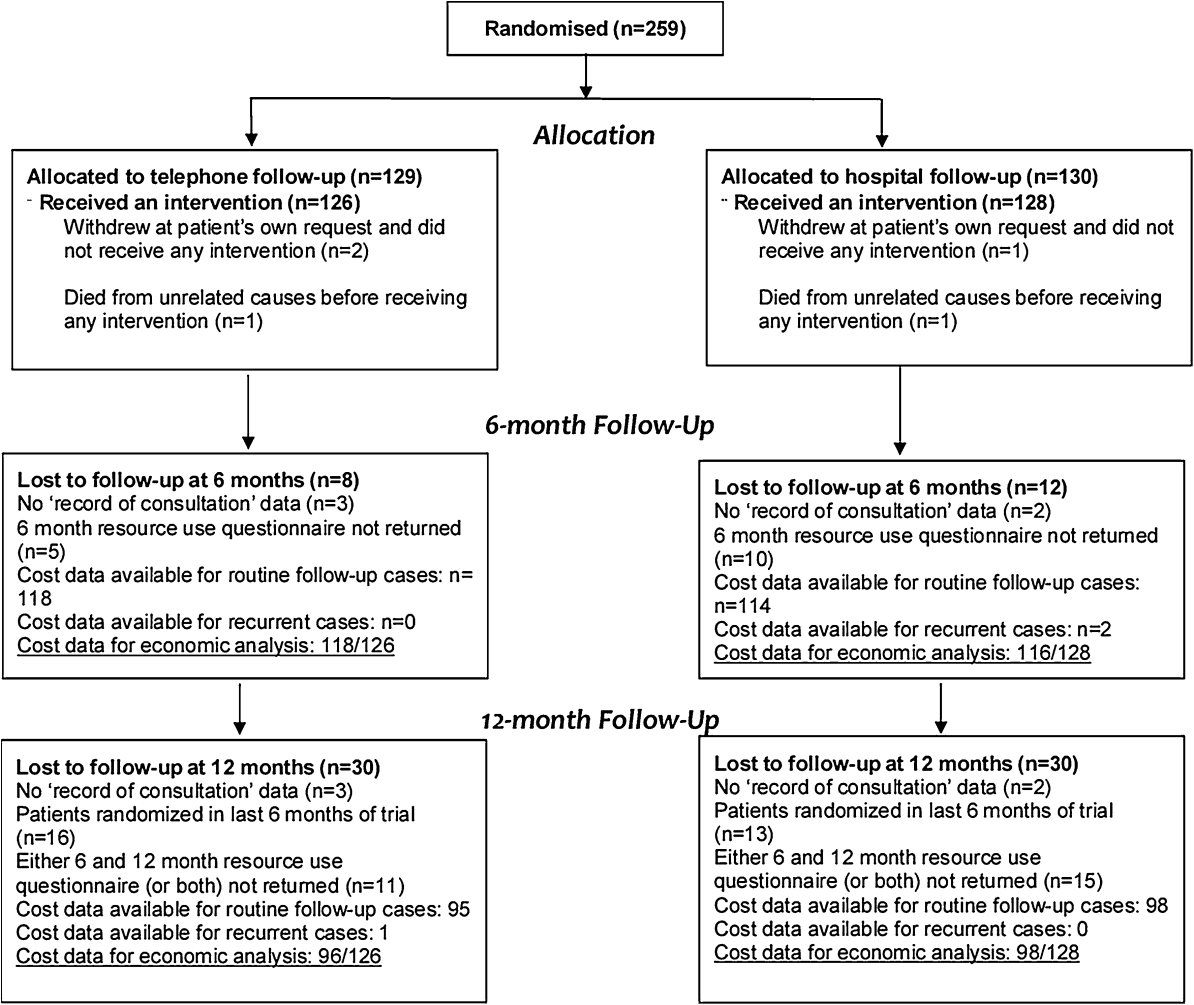
Randomised patients analysed by arm

The ENDCAT trial randomised 259 women on a 1:1 basis between January 2012 and January 2014 at five centres in the north-west of England (Fig. 1). Details of trial methods were published in the study protocol [13] and in the paper describing the results of the trial [14]. In brief, women with a known diagnosis of Stage I endometrial cancer who had completed primary treatment and were attending outpatient clinics for the purposes of routine monitoring and surveillance were eligible for inclusion. No restrictions on age were imposed. Trial participants received either standard hospital outpatient follow-up led by either doctors or nurse specialists (in the control arm) or a telephone follow-up intervention administered by specialist gynaecology oncology nurses.

The trial was designed to assess non-inferiority in the co-primary outcome of psychological morbidity using the state anxiety scale of the State Trait Anxiety Inventory (STAI) [15]. STAI measures an individual’s tendency to anxiety as a stable trait as well as a transitory state. The measure consists of 20 items related to state anxiety (e.g. I am tense) and 20 related to trait (e.g. I feel inadequate). Each item is measured on a 4-point scale ranging from ‘not at all’ to ‘very much so’ for state and ‘almost never’ to ‘almost always’ for trait.

In addition, the ENDCAT trial assessed superiority in terms of the co-primary outcome of patient satisfaction with information received patient satisfaction (measured using questionnaires [14]), and superiority in the secondary outcome of efficiency as measured by cost differences between arms. Patient satisfaction with information was measured by asking participants the following question—‘Did you get all the information you needed at your hospital or telephone appointment?’. Participants were asked to indicate one of five possible responses: ‘I got all/most/some/none of the information I needed’ or ‘I did not need any information’.

The sample size for the randomised controlled trial (RCT) was based on a pre-specified noninferiority margin for the effect of the intervention on the STAI scale. Quality of life was measured using the European Organization for Research and Treatment (EORTC) QLQ-C30 (version 3) instrument [16] and an endometrial cancer-specific module (QLQ-EN24) [17]. Outcomes were measured at baseline and the first post-baseline appointment (which could be between 3 and 12 months after baseline depending on follow-up schedule).

The EORTC QLQ-C30 is a 30-item measure that includes five functional scales (physical, role, cognitive, emotional, social), three symptom scales (fatigue, nausea and vomiting, pain) and six single items; four-point scales range from ‘not at all’ to ‘very much’. An overall general health scale and an overall quality-of-life scale are also included; seven-point scales ranging from ‘very poor’ to ‘excellent’. The EN24 is designed to be used in conjunction with the EORTC QLQ-C30 and consists of 24 items with three functional scales (sexual interest, function and activity) and 10 symptom scales (e.g. hair loss, urological symptoms). Items are rated on a four-point scale ranging from ‘not at all’ to ‘very much’.

More detailed analyses of the ENDCAT trial’s outcomes been reported previously [14]. To undertake a cost-consequence analysis, we compared costs for up to 12 months’ post-randomisation that were associated with each follow-up strategy in the context of the co-primary outcomes of the trial. The costs included those associated with health system resource use, such as nurse and doctor time, and diagnostic tests. This form of analysis, known as cost-consequence analysis, allows decision makers to compare explicitly the costs associated with hospital and telephone follow-up with the outcomes studied in the trial [18].

Healthcare use was assessed using information from a patient-completed resource use questionnaire (RUQ), from case report forms (CRFs), and from reviews of medical notes. A specimen example of the RUQ is included in supplementary material. All RUQs dispatched within ± 14 days of the 6- and 12-month post-randomisation dates were considered valid for analysis. A small number of patients recruited during the final 6 months of the recruitment period were asked to provide only 6 months of follow-up information.

The RUQ (6 and 12 months) consisted of 11 questions related to healthcare use in both primary care [general practitioner (GP) and community nursing services] and secondary care as well as one open question providing an opportunity for comments. Participants were asked to indicate how many times they had contacted a specific healthcare service in the last 6 months; seven options were available ranging from ‘more than once a week’ to ‘never’.

For all participants who attended hospital outpatient appointments a Record of Visit form (CRF) was completed at each attendance and the following information was recorded (if applicable): type of visit (e.g. scheduled, interval), who conducted the consultation, tests and investigations ordered, referrals to other services, indications of recurrent disease, and duration of appointment. For all participants who received telephone consultations, the same information was recorded on a Record of Telephone Consultation form. The Medical Notes Review form enabled a final cross-checking of data on number of appointments (both hospital and telephone), diagnosis of recurrent disease, hospital admissions (related and unrelated to cancer diagnosis) and current status (e.g. continuing on follow-up, discharged, died).

### Training of Nurses for Telephone Follow-Up

Nurses were trained to provide routine telephone follow-up. This training took place in NHS hospital facilities and used no specialised services or capital equipment. The training programme included initial training sessions and a feedback session. The training involved discussions on the rationale for telephone follow-up, skills required to deliver the intervention over the telephone, and a detailed exploration of how to respond to patient information needs.

NHS nurses are remunerated according to ‘bands’ of pay, with higher bands indicating greater seniority and higher pay. Five Band 7 nurses received 6 hours of training from one senior academic nurse (equivalent to Band 8a), and one hour of feedback from two senior academic nurses. Staff attrition and the addition of a new study site required two more nurses (1 Band 8 and 1 Band 6) to receive two hours of training each without a formal feedback session. It was assumed that, in practice, the training would typically be led by a senior nurse (either Band 7 or Band 8a), and we assumed that this nurse would be a Band 8a nurse for the purposes of costing the intervention.

It is possible that nurse training is needed only once to deliver telephone follow-up, and therefore the costs of this training should be amortised over the working lives of nurses receiving the training. The base-case analysis includes training cost over the (relatively short) period of the trial follow-up, while sensitivity analysis excludes training costs from the between-arm cost comparison.

### Sources of Information on Participant Characteristics, Healthcare and Other Costs

Baseline information on age, marital status, employment, education, and occupational grouping was collected using questionnaires. Travel time, mode of transport and patient/companion time off from work for the hospital follow-up appointment were collected from questionnaires completed by patients at the first post-randomisation appointment, and responses were taken to be representative of the patient and family burden for all subsequent appointments. We assumed that employment status did not change during follow-up and that those in employment worked eight hours per day (four hours per day for those working part-time).

Telephone appointments were routinely audio recorded, which allowed for consultation duration to be recorded. The durations of hospital appointments were timed or estimated. Research nurses completed CRFs for clinic visits and telephone consultations during, or shortly after, the consultation. CRFs recorded the duration of the appointment, grade of clinician/band of nurse, referrals, tests or investigations ordered, whether the patients were accompanied on hospital visits, and any signs of recurrence.

Medical notes reviews provided information on cancer-related admissions, recurrence and metastases, date of death and whether death was related to endometrial cancer—these were an alternative source of information on follow-up appointments. Data on other resource use—such as GP visits – were collected from the RUQs sent to patients at 6 and 12 months’ post-randomisation.

### Unit Cost of Resource Items

Unit cost data (Table 1) were drawn primarily from two national sources: the unit costs of health and social care [19] and NHS Reference Costs [20]. The cost of a nurse or doctor contact hour included salary (excluding overtime and shift payments), on-costs (e.g. national insurance contributions), qualifications, the ratio of patient contact to non-contact time, and overheads.

**Table 1:** Unit cost data

Resource	Unit cost in 2012/13 prices (£)	Unit cost in 2016/17 prices (£)^a^	Data source for unit cost valuation	Data source for duration of consultation used in cost calculations
Nurse training for telephone consultation				
Nurse training, including feedback sessions	2958.95	3143.00	UCHSC^d^ 2013	
Consultations				
Telephone consultations				
Band 7 nurse, per patient-related hour	70.02	74.38	UCHSC 2013	Data collected in trial
Band 8a nurse, per patient-related hour	78.49	83.37	UCHSC 2013	Data collected in trial
Cost of landline phone call per minute	0.09	0.10	BT^b^	Data collected in trial
Hospital consultations				
Consultant doctor, per patient-related hour	246.31	261.63	UCHSC 2013	Data collected in trial
Registrar doctor, per patient-related hour	106.07	112.67	UCHSC 2013	Data collected in trial
F2/FI: Average of Foundation year 2 and year 1, per patient-related hour	65.86	69.96	UCHSC 2013	Data collected in trial
Nurse specialist, per patient-related hour	103.85	110.31	UCHSC 2013	Data collected in trial
Private car rates per mile	0.45	0.48	HMRC^b^	
Hospital parking per consultation	2.07	2.20	Hospital data	
Median 4-h wage of patient	46.48	49.37	Annual Survey of Hours and Earnings [34]	
Hospital transport services	13.58	14.42	Liu et al. [35]	
Tests (HRG code)^c^				
Computerised tomography (RA08A-RA14Z)	106.38	113.00	Reference Costs 2012/13^c^	
Ultrasonography (RA23Z-RA24Z)	51.88	55.11	Reference Costs 2012/13	
Diagnostic hysteroscopy with biopsy (MA32Z)	846.25	898.89	Reference Costs 2012/13	
Mid-stream urine sample (DAPS04)	1.25	1.33	Reference Costs 2012/13	
Blood tests (DAPS03)	1.94	2.06	Reference Costs 2012/13	
Wound swab/bile culture test/histology/high vaginal swab (DAPS02)	38.74	41.15	Reference Costs 2012/13	
Referrals (HRG code)				
Community-based physiotherapist per-patient related hour	75.11	79.78	UCHSC 2013	Based on 30-min consultation, UCHSC 2010^e^
Complementary therapies, not otherwise specified—costed as non-admitted, non-consultant attendance (WF01A-WF02D)	62.93	66.84	Reference Costs 2012/13	
GP consultation at practice	45.00	47.80	UCHSC 2013	Based on 11.7-min consultation, UCHSC 2013
Hospital-based physiotherapist per patient-related hour	79.87	84.84	UCHSC 2013	Based on 23.3-min consultation, UCHSC 2010
Multi-disciplinary teams (CMDT_SpG)	112.49	119.49	Reference Costs 2012/13	
Psychologist/counsellor per patient-related hour	131.40	139.57	UCHSC 2013	Based on assumed duration of 60 min
Radiologist per patient-related hour	36.72	39.00	UCHSC 2013	Based on 20-min consultation, UCHSC 2010
Urodynamics (LB42A)	396.69	421.37	Reference Costs 2012/13	
Other healthcare used during follow-up				
Community-based occupational therapy per patient-related hour	75.13	79.80	UCHSC 2013	Based on 40-min consultation, UCHSC 2010
District nurse per patient-related hour	69.97	74.32	UCHSC 2013	Based on 20-min consultation, UCHSC 2010
GP home visit	114.00	121.09	UCHSC 2013	Based on 23.4-min consultation, UCHSC 2013
GP phone consultation	27.00	28.68	UCHSC 2013	Based on 7.1-min consultation, UCHSC 2013
Hospital genetics (WF01A-WF01D)	193.74	205.79	Reference Costs 2012/13	
Hospital incontinence clinic	139.23	147.89	UCHSC 2013	Based on assumed duration of 60 min, costed as the time of one specialist hospital-based nurse
Hospital occupational therapy per patient-related hour	79.84	84.81	UCHSC 2013	Based on 30-min consultation, UCHSC 2010
Hospital pain team (AB03Z-AB06Z)	175.91	186.85	Reference Costs 2012/13	
Hospital secretary/receptionist per contracted hour	33.67	35.76	UCHSC 2013	Based on assumed duration of 15 min
Mental health services	136.00	144.46	UCHSC 2013	
Other community specialist nurse per patient-related hour	62.85	66.76	UCHSC 2013	Based on assumed duration of 15 min
Practice nurse per patient-related hour	51.49	54.69	UCHSC 2013	Based on 15.5-min consultation, UCHSC 2010
Walk-in centre	41.96	44.57	UCHSC 2011	

^a^2016/17 prices based on UK GDP deflator at market prices

^b^BT: BT Group plc, the major telephone landline provider in the UK. HMRC: Her Majesty’s Revenue and Customs

^c^If sufficient detail was not available from medical notes, the averages of ‘Currency Code’ costs from NHS Reference Costs were calculated by weighting the total number of finished consultant episodes across all service descriptions

^d^UCHSC 2013 refers to [19]. UCHSC 2011 refers to [36]. UCHSC 2010 refers to [37]

^e^When UCHSC was used as the source of data for the duration of a typical appointment, the most recently available data were used where possible

We collected unit cost data from 2012/13 as this was approximately the mid-point of trial recruitment. Unit costs from this period therefore reflect the structure of relative costs prevailing within the NHS at the time of the trial. We inflated unit costs from the time of the trial using the most recent (2016/17) UK GDP deflator [21] (an established measure of general inflation) so that costs are expressed in pounds sterling at 2016/17 prices. Other details on the construction of unit costs are provided in online supplementary material.

### Perspective

The primary economic analysis took a health system (i.e. NHS) perspective for costs and focussed on routine monitoring and surveillance costs. The perspective of an economic evaluation establishes which costs should be measured, how these costs should be valued, and how they should be related to the consequences of interest. A health-system perspective is most relevant to those involved in funding and commissioning health services.

The time horizon for the economic analysis was up to 12 months. Costs were not discounted. We excluded costs known to be incurred post-recurrence, or collected from RUQs returned post-recurrence, on the grounds that time until recurrence detection is not causally related to the mode of routine follow-up [22, 23]. In secondary analysis we examined healthcare costs associated with recurrence and estimated personal (e.g. travel) and productivity (i.e. time off work) costs.

### Analysis Sample

Three women (2 telephone arm, 1 hospital arm) withdrew from the trial at their own request and provided no resource-use follow-up data. Two women (1 in each arm) died from causes unrelated to endometrial cancer before they received any follow-up or provided resource-use data. These five patients were excluded from all economic analyses. We analysed the remaining patients in the groups to which they were randomised.

Analysis was conducted on available cases. Patients were eligible for inclusion in the six-month analysis if they had returned the 6-month RUQ and had undergone at least one follow-up consultation. Patients were eligible for inclusion in the 12-month analysis if they underwent at least one follow-up consultation and returned both 6- and 12-month RUQs (Fig. 1).

### Missing Data

Missing data were infrequent (Fig. 1), and logistic regression analysis confirmed that “missingness” was not associated with allocation at 6 or 12 months. Missing responses to specific resource-use questions on otherwise complete RUQs were taken as null responses, i.e. a response that the resource in question had not been used.

### Subgroup Analysis

The pre-specified subgroups were aligned to the subgroup variables used in the ENDCAT clinical effectiveness analysis [14]: routine follow-up interval at recruitment (< 6; ≥ 6 months); age (< 70; ≥ 70) years; level of education (no qualification; some qualification but no degree; degree holder); work status at recruitment (actively working; not actively working); and occupational group (managerial/professional; administration/skilled/trades/caring/sales; operatives/elementary occupations).

### Inference

Boot-strapped bias-corrected and accelerated (BCA) percentile 95% confidence intervals (CI) using 10,000 replications were calculated in Stata version 15 (Stata Corp: College Station, TX, USA). Follow-up costs were assessed in two ways. The first examined whether there was a difference in mean costs associated with consultations, tests and referrals made during routine follow-up (‘follow-up’ costs). The second approach examined whether there were differences in mean costs associated with all other resource use (e.g. GP visits) recorded in the trial (‘total costs’). For each approach, percentile 95% CI around mean differences in cost between arms were constructed. All inferential analysis was conducted using Stata version 15.

### Univariate Sensitivity Analysis

We examined the effect of increasing the salaries for doctors and nurses to include remuneration for shift work and overtime [19]. We also examined the effects of excluding the costs of nurse training from the telephone arm, which could be considered a routine part of continuing nursing education, and which will likely be lower (per-patient) outside of the trial setting.

## Results

### RCT Results

Participant characteristics were similar between arms. The mean age of participants at randomisation was 65 years. The median time from diagnosis for participants at randomisation was 12 months. Some 63% of women were receiving routine follow-up at either 3- or 4-monthly intervals, 32% at 6-monthly intervals, and 5% at annual intervals. Other information on participant characteristics is provided in Beaver et al [14].

Patient-reported anxiety at follow-up was slightly lower in the telephone arm and within the pre-specified non-inferiority limit of − 3.5 (adjusted mean difference 0.7, 95% CI on an intention-to-treat basis: − 1.9 to 3.3) [14]. There was no evidence of a difference between groups in satisfaction with information received (adjusted odds ratio for lesser satisfaction in hospital group relative to telephone group: 0.9, 95% CI 0.40 to 2.41). However, more participants in the hospital group than the telephone group (27·8 vs. 13·5%) stated that they did not need any information (*p* = 0.003).

There were no differences between arms in quality of life; this was assessed by a comparison between arms of each subscale of both EORTC QLQ-C30 instrument (15 subscales/items) and the QLQ-EN24 endometrial cancer module (13 subscales/items). The only instance of a between-group difference in either instrument was that hospital follow-up participants were slightly more likely to report constipation (*p* = 0.035) in QLQ-C30. No significant between-arm differences in physical or psychosocial needs or time to detection of recurrent disease were reported.

### NHS Costs after 6 and 12 Months

More routine consultations took place in the telephone arm at six and 12 months. In the hospital arm most of the consultations at 6 months (Table 2) were with a consultant (46/121) or registrar (45/121).

**Table 2:** Cost of routine follow-up at 6 months post-randomisation

	Telephone follow-up (*n* = 118 available cases)		Hospital follow-up (*n* = 116 available cases)		Mean difference (95% BCA percentile CI)
	*N*	Mean cost per patient (£)	*N*	Mean cost per patient (£)	
Telephone consultations	123	19.46	0	–	
Hospital consultations	14	4.39	121	25.97	
Nurse training cost per telephone consultation		25.56			
Tests^a^	4	10.00	8	3.97	
Referrals^b^	16	9.19	3	4.30	
Other healthcare use during period of follow up		365.40		391.95	
Total health system costs		434		426	£8 (− £147 to £141)

Totals health system costs are reported to nearest £1, and may not sum due to rounding

*BCA* bias-corrected and accelerated, *CI* confidence interval, *CT* computed tomography, *GP* general practitioner

^a^The four tests in the telephone arm, and their associated frequencies, were as follows: CT (2), ultrasonography (1), biopsy (1). The eight tests in the hospital arm, and associated frequencies, were as follows: CT (2), ultrasonography (2), biopsy (1), histology (1), blood sample (1), abdominal wound swab (1), bile swab (1)

^b^The 16 referrals in the telephone arm, and their associated frequencies, were as follows: GP (6), hospital doctor (2), consultant (2), psychologist (3), physiotherapist (1), complementary therapies (2). The 3 referrals in the hospital arm, and their associated frequencies, were as follows: hospital doctor (1), physiotherapist (1), and urodynamic therapies (1)

Hospital consultations that took place for patients randomised to telephone follow-up were generally not routine scheduled follow-up appointments, were conducted only by senior doctors, and took longer than the consultations undertaken by equivalent grades of staff in the hospital arm. Similar patterns were observed at 12 months (Table 3). Telephone follow-up was not superior in cost terms at either 6 or 12 months.

**Table 3:** Cost of routine follow-up at 12 months post-randomisation

	Telephone follow-up (*n* = 96 available cases)		Hospital follow-up (*n* = 98 available cases)		Mean difference (95% BCA percentile CI)
	*N*	Mean cost per patient (£)	*N*	Mean cost per patient (£)	
Telephone consultations	214	37.27	2	0.32	
Hospital consultations	17	6.25	200	47.45	
Nurse training cost per telephone consultation		14.41		0.14	
Tests^a^	6	11.88	13	24.64	
Referrals^b^	15	9.83	4	5.47	
Other healthcare use during period of follow up		666.00		744.60	
Total health system costs		746		823	– £77 (– £334 to £154)

Totals and subtotals are reported to nearest £1, and may not sum due to rounding

*BCA* bias-corrected and accelerated, *CI* confidence interval, *CT* computed tomography, *GP* general practitioner

^a^The six tests in the telephone arm were one each of CT, ultrasonography, biopsy, histology, high vaginal swab, and mid-stream urine sample. The 13 tests in the hospital arm, and associated frequencies, were as follows: CT (3), ultrasonography (2), biopsy (2), histology (1), high vaginal swab (3), mid-stream urine sample (1), blood sample (1)

^b^The 15 referrals in the telephone arm, and their associated frequencies, were as follows: GP (6), hospital doctor (2), consultant (3), psychologist (2), physiotherapist (1), complementary therapies (1). The 4 referrals in the hospital arm, and their associated frequencies, were as follows: GP (1), physiotherapist (1), radiology (1), and urodynamic therapies (1)

Mean consultation duration was longer in the telephone arm (12.5 min) than in the hospital arm (8.9 min). Consultants and registrars were responsible for 100% (6 months) and 88% (12 months) of all hospital-based consultations in the telephone follow-up arm, compared to 75% (6 months) and 80% (12 months) of consultations in patients randomised to hospital follow-up. More referrals were made or ordered in the telephone arm.

At 6 months, mean routine follow-up costs in the telephone arm were higher than in the hospital arm. Nurse training costs accounted for approximately 75% of the difference in follow-up costs at 6 months. This is a consequence of the fixed cost of nurse training being spread over a modest number of calls. The lower unit cost of nurse-led telephone consultations is counterbalanced by the greater number and longer duration of routine consultations. Mean per patient routine follow-up costs at 12 months in the telephone arm were slightly higher than in the hospital arm.

These cost differences are summarised against the co-primary outcomes of the ENDCAT trial in the cost-consequences table (Table 4).

**Table 4:** Summary of costs and consequences

Costs	Between-group difference (point estimate)	95% BCA CI for between-group difference
Total health system costs at 6 months	£8	(− £147 to £141)
Total health system costs at 12 months	− £77	(− £334 to £154)

Consequences—*STAI*^*a*^	Between-group difference (point estimate)	95% CI for between-group difference
STAI—analysis ‘adjusted for treatment received’—for participants with 6 month health economic data	0.7	− 1.9 to 3.3
STAI – analysis ‘adjusted for treatment received’—for participants with 12-month health economic data	0.6	− 2.4 to 3.5

Consequences—satisfaction with information received^b^	Odds ratio (OR)	95% CI for OR
Satisfaction with information received—analysis for participants with 6-month health economic data	0.8	0.4–1.9
Satisfaction with information received—analysis for participants with 12-month health economic data	0.6	0.2–1.7

*BCA* bias-corrected and accelerated, *CI* confidence interval, *STAI* State Trait Anxiety Inventory

^a^The primary statistical analysis of the psychological morbidity outcome was based on a complier-adjusted causal analysis—see [14] for further details

^b^Those responding “I did not need any information” in response to satisfaction with information received were classified as missing for this analysis

### Univariate Sensitivity Analysis of Costs at 6 and 12 Months

The cost comparison at 6 months is affected by the high fixed cost of nurse training. Excluding the costs of nurse training from the telephone arm lowers the estimated mean difference per patient in total costs to − £18 (95% BCA CI: − £170 to £118) at 6 months, and at 12 months to − £91 (95% CI: − £347 to £146).

There was little impact on the results of including the costs of payments for overtime and shift work for doctors and nurses. The difference in mean total costs was similar to the base case at both 6 months (mean difference £10: 95% BCA CI − £146 to £146), and 12 months (mean difference − £72: 95% BCA CI: − £346 to £167).

### Patient Productivity and Transport Costs

A higher proportion of patients in the hospital arm (21/110; 19.1%, Table 5) reported taking time-off work for the appointment than in the telephone arm (3/111; 2.7%; Fisher’s exact test *p* < 0.001). More than half of those randomised to the hospital follow-up arm indicated that they were accompanied by a companion, 12.7% of whom took time off work. Information on whether patients participating in telephone consultations were accompanied was not recorded.

**Table 5:** Patient and companion time for hospital consultations

	Telephone consultation		Hospital consultation	
	*N*	Response	*N*	Response
Presence of companion?	N/A^a^	N/A^a^	111 responses	Yes—61 (55.0%) No—50 (44.0%)
Time off work for the appointment^b^				
Patient	111 responses	Yes—3 (2.7%) No—108 (97.3%)	110 responses	Yes—21 (19.1%) No—89 (80.9%)
Companion	N/A^a^	N/A^a^	63 responses	Yes—8 (12.7%) No—55 (87.3%)

^a^Patients were asked whether they had been accompanied to a hospital consultation only. Any accompaniment for telephone consultations that may have taken place was not recorded during the trial

^b^Two responses to this question indicated that no time off work had actually been taken because of compensating shift changes or the time being made up in other ways. These two patients were assumed to have incurred no productivity cost, which is a conservative assumption

Average return journey travel costs for attendance at hospital clinics were estimated to be £11.47 per appointment, excluding the costs of hospital transport which we assume is a cost to the NHS rather than a direct patient cost. More details are provided in Supplementary Table A1 in the supplementary material.

The numbers of patients reporting information relating to productivity costs was very small: *n* = 3 patients in the telephone arm, and *n* = 19 patients in the hospital arm. More detail is provided in Supplementary Table A2 in supplementary material. Mean costs were lower per patient in the telephone consultation arm (£29) than in the hospital consultation arm (£57) but inference cannot be reliably conducted given the small number of individuals reporting data.

### Subgroup Analysis

The results of the subgroup analysis are reported in Supplementary Table A3 in the supplementary material. Statistical power to detect between-arm differences is very low, and the evidence available from this analysis does not offer strong evidence of cost differences amongst the pre-specified subgroups.

### National Health Service Costs of Recurrent and Metastatic Cases

Two women (in the hospital arm) with resource use data at 6 months had recurrence/metastases during this period, and one (in the telephone arm) with resource use data at 12 months had recurrence/metastases between 6 and 12 months. All three women underwent some routine follow-up.

In the six-month sample, recurrence was diagnosed 100 and 173 days from randomisation. Mean cost for healthcare deemed to relate only to recurrence was £5597. In the 12-month sample, recurrence was diagnosed at 308 days after randomisation. This patient is estimated to have incurred costs of £18,290 associated with relatively heavy use of a wide variety of different resources.

## Discussion

### Main Findings

Telephone follow-up was estimated to be cost-neutral, but not cost saving, for the NHS. Superiority of telephone follow-up with respect to health system costs was not demonstrated. There was some evidence that it may be more efficient for patients and wider society.

### Reasons for Similarity of NHS Costs

Why were NHS costs not lower in the telephone arm? One reason is that the mean durations of consultation were longer in the telephone arm, possibly because of the use of a script for these consultations, and the effects of allocating 20 min for telephone follow-up calls compared to the 10 min scheduled for routine hospital clinic visits. The telephone intervention was delivered by clinical nurse specialists within the confines of current staffing levels and did not take staff away from their usual duties or introduce new staff to deliver the intervention. Although a period of training was required, and this was included as a cost in the base-case analysis, in clinical practice this training could be incorporated as part of continuing professional development.

Mean differences in cost are sensitive to training cost, and to remuneration of doctors and nurses involved in hospital follow-up, which in practice will be somewhere between basic salary (included in the main results) and supplemented income (included in sensitivity analysis). We reflected the lower capital and overhead costs involved in providing telephone follow-up, although this might understate the costs savings if lower numbers of hospital visits lead to more efficient long-term hospital capital planning.

More referrals were made or ordered in the telephone arm. This finding was also observed in a similar economic evaluation for breast cancer patients [22]. A study on nurse-led follow-up for prostate cancer patients also indicated that nurses made more referrals than their medical colleagues [24]. The possibility of a greater number of referrals when nurses substitute for doctors was noted in a more general evaluation of advanced nursing roles in 12 developed countries [25].

The structured nature of the intervention in the ENDCAT trial encouraged patients to voice their information needs and concerns. Hospital doctors in busy hospital clinics do not tend to follow a structured protocol for the consultation and appointments are brief [4]. It could be argued that the nurses in the telephone arm were responsive to patients’ needs and concerns and made appropriate referrals. Alternatively, the nurses were inexperienced at delivering the telephone intervention at the outset and may have made additional referrals in order to ensure they did not miss anything. As most referrals in the telephone arm were made to a GP, issues may have arisen that were unrelated to the cancer diagnosis and were in response to more general questions about changes in condition.

Fewer patients reported taking time off work for the telephone consultation, while more than half of patients had a companion present at hospital consultations. The average return journey hospital travel cost of approximately £11 will be incurred for every hospital consultation. Travel and productivity costs were not collected for each consultation, which means that changes in patient circumstances were not captured. Telephone follow-up was associated with less likelihood of delay in undergoing the consultation; intervention patients reported high levels of satisfaction with the timeliness of their consultations [14].

### Strengths

The ENDCAT trial provides the only findings published [14] to date of an evaluation of hospital versus telephone follow-up for patients with endometrial cancer. The design of the intervention evaluated in the trial, and the subject of the economic analysis described in this study, built on evidence generated by previous examinations of nurse-led telephone follow-up [26, 27].

The economic analysis adds to the limited evidence base (discussed below) on the cost implications of telephone follow-up after treatment for cancer. The economic analysis was based on a prospective analysis of detailed patient-level data, and encompassed elements of health system, personal and societal perspectives. The trial was conducted in five centres in the north-west of England, and there is no obvious reason why the findings could not be generalizable to other NHS contexts. The implications for other countries are less clear, and will depend on the frequency of routine follow-up, the pressure on hospital clinic space, and the seniority of staff involved in hospital-based follow-up.

### Limitations

The study was not powered to detect differences in cost, and the failure to reject the null hypothesis of no difference in healthcare cost may reflect a Type II error. However, the relatively narrow confidence interval around the point estimate for differences in routine NHS costs at 12 months suggests that nurse-led telephone follow-up was approximately cost-neutral.

The economic analysis was limited to one year from randomisation, which does not reflect the full duration of follow-up that women will undergo in recovering from endometrial cancer. However, trial participants were at various stages post-diagnosis, with a median of 12 months (Interquartile range: 4 to 24 months). Some 63% of women were receiving routine follow-up at either 3- or 4-monthly intervals, 32% at 6-monthly intervals, and 5% at annual intervals. The 12-month period of *trial* follow-up may therefore reflect the experiences of women at different stages of *clinical* follow-up, but the evidence presented here is necessarily an approximation to—rather than a direct quantitative characterisation of—longer-term outcomes.

The design of the trial meant that disease-specific quality-of-life data were collected post-randomisation only at the first post-baseline appointment, the timing of which depended on whether participants were on a 3-monthly, 6-monthly or annual follow-up schedule. The economic analysis was therefore restricted to a cost-consequence analysis, which is necessarily narrower in scope than alternative study designs that—for example—measure disease-specific or generic measures of quality of life at baseline and all subsequent follow-up time points. Recall bias may have affected self-report of resource use not collected from medical records, although this bias may have affected each arm in a similar fashion.

We do not have information on whether companions took time off work or incurred other costs to be present during telephone consultations. However, we consider that any effect on costs is likely to be modest.

### Other Evidence

The amount of evidence available for cost analysis of similar interventions in endometrial and other cancers is limited [28], although there is a growing literature in nurse follow-up after treatment for breast cancer [29]. A UK trial [26] comparing nurse-led telephone follow-up for patients recovering from breast cancer to routine hospital follow-up also observed longer and more frequent consultations in the telephone arm. Expensive telephone consultation costs, and the use of junior doctors in the hospital arm, meant that NHS costs were significantly higher in the telephone arm, although patients randomised to telephone follow-up also reported lower travel and productivity costs.

A trial in Sweden [30] found the costs of on-demand and scheduled nurse follow-up to be 20% lower, and significantly so, than routine follow-up after treatment for breast cancer. The results were also sensitive to the costs of telephone consultations. A Dutch economic evaluation [31] of four follow-up strategies after treatment for breast cancer, involving combinations of educational programmes in conjunction with nurse-led and telephone follow-up, observed modest cost-differences but found that nurse-led follow-up combined with an educational programme was cost-effective. A systematic review of breast cancer follow-up [29] found that considerations pertaining to the design of follow-up, such as frequency, can influence cost differences.

### Summary Implications for Policy Makers

Nurse-led telephone follow-up is an effective alternative for patients at low risk of recurrence. Nurse specialists are limited in number and are unlikely to have the resources to provide follow-up care to all patients diagnosed with low-risk cancers. However, a survey of the adult cancer workforce reported that oncology nurses perceived that they had the skills to manage long-term follow-up [32]. Nurse-led telephone follow-up is therefore supported by clinical evidence of effectiveness, has a workforce capable of delivering the intervention, and is cost-neutral for the NHS. A reduction in the number of outpatient appointments with more self-management approaches and patient initiated follow-up services is currently being advocated [33]. However, until these initiatives are fully implemented, telephone follow-up presents providers and commissioners of services with a viable alternative, freeing up doctor time and clinic space.

## Conclusion

Overall, the evidence from the ENDCAT trial suggests that there may be an economic case for considering the use of telephone follow-up for this patient group. The intervention is not more expensive than hospital follow-up at 12 months, is associated with lower personal and productivity costs, and is not associated with psychological detriment.

## Electronic supplementary material

Below is the link to the electronic supplementary material.

Supplementary material 1 (PDF 104 kb)

Supplementary material 2 (DOC 74 kb)

## References

[1] Maddams J, Utley M, Moller H (2012). Projections of cancer prevalence in the United Kingdom, 2010–2040. Br J Cancer.

[2] Maher J, McConnell H (2011). New pathways of care for cancer survivors: adding the numbers. Br J Cancer.

[3] Lajer H, Jensen MB, Kilsmark J, Albaek J, Svane D, Mirza MR (2010). The value of gynecologic cancer follow-up: evidence-based ignorance?. Int J Gynecol Cancer.

[4] Jefford M, Rowland J, Grunfeld E, Richards M, Maher J, Glaser A (2013). Implementing improved post-treatment care for cancer survivors in England, with reflections from Australia, Canada and the USA. Br J Cancer.

[5] Pagh A, Vedtofte T, Lynggaard CD, Rubek N, Lonka M, Johansen J (2013). The value of routine follow-up after treatment for head and neck cancer. A National Survey from DAHANCA. Acta Oncol.

[6] NHS England. Achieving world class cancer outcomes: taking the strategy forward. 2016. https://www.england.nhs.uk/wp-content/uploads/2016/05/cancer-strategy.pdf. Accessed 4 Apr 2018.

[7] Tjalma WAA, Van Dam PA, Makar AP, Cruickshank DJ (2004). The clinical value and the cost-effectiveness of follow-up in endometrial cancer patients. Int J Gynecol Cancer..

[8] Stewart B, Wild C, editors. World Cancer Report 2014: International Agency for Research on Cancer. Lyon: World Health Organization; 2014.

[9] Plataniotis G, Castiglione M, On behalf of the EGWG (2010). Endometrial cancer: ESMO Clinical Practice Guidelines for diagnosis, treatment and follow-up. Ann Oncol.

[10] Creasman WT, Odicino F, Maisonneuve P, Quinn MA, Beller U, Benedet JL (2006). Carcinoma of the corpus uteri. FIGO 26th Annual Report on the Results of Treatment in Gynecological Cancer. Int J Gynaecol Obstet.

[11] Amant F, Moerman P, Neven P, Timmerman D, Van Limbergen E, Vergote I (2005). Endometrial cancer. Lancet..

[12] Jeppesen MM, Mogensen O, Hansen DG, Iachina M, Korsholm M, Jensen PT (2017). Detection of recurrence in early stage endometrial cancer—the role of symptoms and routine follow-up. Acta Oncol..

[13] Beaver K. Comparing hospital and telephone follow up for women treated for endometrial cancer (ENDCAT): ENDometrial CAncer Telephone follow up trial). ISRCTN Trial Registry 2011. 10.1186/ISRCTN75220876.

[14] Beaver K, Williamson S, Sutton C, Hollingworth W, Gardner A, Allton B (2017). Comparing hospital and telephone follow-up for patients treated for stage–I endometrial cancer (ENDCAT trial): a randomised, multicentre, non-inferiority trial. BJOG Int J Obstet Gynaecol..

[15] Spielberger C (1983). State-trait anxiety inventory.

[16] Aaronson NK, Ahmedzai S, Bergman B, Bullinger M, Cull A, Duez NJ (1993). The European organization for research and treatment of cancer QLQ-C30: a quality-of-life instrument for use in international clinical trials in oncology. J Natl Cancer Inst.

[17] Greimel E, Nordin A, Lanceley A, Creutzberg CL, van de Poll-Franse LV, Radisic VB (2011). Psychometric validation of the European Organisation for Research and Treatment of Cancer Quality of Life Questionnaire-Endometrial Cancer Module (EORTC QLQ-EN24). Eur J Cancer.

[18] Coast J. Is economic evaluation in touch with society’s health values? BMJ. 2004;329:1233–6. 10.1136/bmj.329.7476.1233 PMC529373 15550430

[19] Curtis L (2013). Unit costs of health and social care 2013.

[20] Department of Health. NHS Reference Costs 2012/13. London; 2013. https://www.gov.uk/government/publications/nhs-reference-costs-2012-to-2013. Accessed 5 Apr 2018.

[21] HM Treasury. GDP deflators at market prices, and money GDP November 2017 (Autumn Budget 2017). 2017 03/01/2018]; Available from: https://www.gov.uk/government/statistics/gdp-deflators-at-market-prices-and-money-gdp-november-2017-autumn-budget-2017.

[22] Nicolaije KAH, Ezendam NPM, Vos MC, Boll D, Pijnenborg JMA, Kruitwagen RFPM (2013). Follow-up practice in endometrial cancer and the association with patient and hospital characteristics: a study from the population-based PROFILES registry. Gynecol Oncol..

[23] Vistad I, Moy BW, Salvesen HB, Liavaag AH (2011). Follow-up routines in gynecological cancer—time for a change?. Acta Obstet Gynecol Scand.

[24] Helgesen F, Andersson SO, Gustafsson O, Varenhorst E, Goben B, Carnock S (2000). Follow-up of prostate cancer patients by on-demand contacts with a specialist nurse: a randomized study. Scand J Urol Nephrol.

[25] Delamaire M, Lafortune G. Nurses in Advanced Roles. OECD Health Working Papers. 2010; No 54.

[26] Beaver K, Tysver-Robinson D, Campbell M, Twomey M, Williamson S, Hindley A, et al. Comparing hospital and telephone follow-up after treatment for breast cancer: randomised equivalence trial. BMJ. 2009;338:a3147. 10.1136/bmj.a3147 PMC2628299 19147478

[27] Beaver K, Campbell M, Williamson S, Procter D, Sheridan J, Heath J (2012). An exploratory randomized controlled trial comparing telephone and hospital follow-up after treatment for colorectal cancer. Colorectal Dis.

[28] Dickinson R, Hall S, Sinclair JE, Bond C, Murchie P (2014). Using technology to deliver cancer follow-up: a systematic review. BMC Cancer..

[29] van Hezewijk M, Elske van den Akker M, van de Velde Velde CJH, Scholten AN, Hille ETM (2012). Costs of different follow-up strategies in early breast cancer: a review of the literature. Breast..

[30] Koinberg I, Engholm GB, Genell A, Holmberg L (2009). A health economic evaluation of follow-up after breast cancer surgery: Results of an rct study. Acta Oncol.

[31] Kimman ML, Dirksen CD, Voogd AC, Falger P, Gijsen BCM, Thuring M (2011). Economic evaluation of four follow-up strategies after curative treatment for breast cancer: results of an RCT. Eur J Cancer..

[32] Faithfull S, Samuel C, Lemanska A, Warnock C, Greenfield D (2016). Self-reported competence in long term care provision for adult cancer survivors: a cross sectional survey of nursing and allied health care professionals. Int J Nurs Stud.

[33] Department of Health, Macmillan Cancer Support & NHS Improvement. Living with and beyond cancer: taking action to improve outcomes (an update to the 2010 The National Cancer Survivorship Initiative Vision). London; 2013.

[34] Office of National Statistics. Annual Survey of Hours and Earnings, 2013 Provisional Results. Available from: http://webarchive.nationalarchives.gov.uk/20160107081242/, http://www.ons.gov.uk/ons/rel/ashe/annualsurvey-of-hours-and-earnings/2013-provisional-results/index.html. Accessed 5 Apr 2018.

[35] Liu F, Treharne C, Culleton B, Crowe L, Arici M (2014). The financial impact of increasing home-based high dose haemodialysis and peritoneal dialysis. BMC Nephrol.

[36] Curtis L (2011). Unit costs of health and social care 2011.

[37] Curtis L (2010). Unit costs of health and social care 2010.

