# Supplementary material for: Cost-Consequence Analysis Alongside a Randomised Controlled Trial of Hospital Versus Telephone Follow-Up after Treatment for Endometrial Cancer
Source: Appl Health Econ Health Policy. 2018 Apr 12;16(3):415–27. doi: 10.1007/s40258-018-0378-6 (PMC5940716; doi:10.1007/s40258-018-0378-6)
Supplement: Supplementary file 1 — Supplementary material 1 (PDF 104 kb) [file 40258_2018_378_MOESM1_ESM.pdf]

Form I

ID Number:

ENDCAT: ENDOMETRIAL CANCER TELEPHONE FOLLOW-UP TRIAL

6 months health care appointments questionnaire

Thank you for continuing to take part in the study that compares telephone and hospital follow-up for women treated for endometrial (womb) cancer. The following questions are about the health care you have had over the last six months. Please answer the following questions as honestly as you can; there are no right or wrong answers. This questionnaire is quite confidential – it will not be shown to the doctors or nurses involved in your care.

GP and community nursing services

In the last six months have you been in touch with any of the following people? (Please tick the most appropriate box for each question).

Service	Number of times in last 6 months
1. Number of times you had an appointment with a GP (family doctor) at the surgery	☐ More than once a week (about 50 times)<br>☐ About once a week (about 25 times)<br>☐ About once every other week (about 12 times)<br>☐ About once every month (about 6 times)<br>☐ Less than once a month (about 3 times)<br>☐ Once or twice<br>☐ Never
2. Number of times you had an appointment with a GP (family doctor) at your home	☐ More than once a week (about 50 times)<br>☐ About once a week (about 25 times)<br>☐ About once every other week (about 12 times)<br>☐ About once every month (about 6 times)<br>☐ Less than once a month (about 3 times)<br>☐ Once or twice<br>☐ Never
3. Number of times you spoke to a GP (family doctor) on the telephone	☐ More than once a week (about 50 times)<br>☐ About once a week (about 25 times)<br>☐ About once every other week (about 12 times)<br>☐ About once every month (about 6 times)<br>☐ Less than once a month (about 3 times)<br>☐ Once or twice<br>☐ Never

Form I

Service	Number of times in last 6 months
4. Number of times you had an appointment with a practice nurse at the surgery	☐ More than once a week (about 50 times)<br>☐ About once a week (about 25 times)<br>☐ About once every other week (about 12 times)<br>☐ About once every month (about 6 times)<br>☐ Less than once a month (about 3 times)<br>☐ Once or twice<br>☐ Never
5. Number of times you had an appointment with a district nurse at your home	☐ More than once a week (about 50 times)<br>☐ About once a week (about 25 times)<br>☐ About once every other week (about 12 times)<br>☐ About once every month (about 6 times)<br>☐ Less than once a month (about 3 times)<br>☐ Once or twice<br>☐ Never
6. Number of times you had contact with any other community health services (e.g. physiotherapist, health visitor, cousellor)<br><br>Please say who you contacted<br><br>__________________________	☐ More than once a week (about 50 times)<br>☐ About once a week (about 25 times)<br>☐ About once every other week (about 12 times)<br>☐ About once every month (about 6 times)<br>☐ Less than once a month (about 3 times)<br>☐ Once or twice<br>☐ Never

Form I

Hospital services
In the last six months have you been in touch with any of the following people? (Please tick the most appropriate box for each question).

Service	Number of times in last 6 months
7. Number of times you had an appointment with a hospital doctor for something that was NOT related to your diagnosis/ treatment of endometrial (womb) cancer	☐ More than once a week (about 50 times)<br>☐ About once a week (about 25 times)<br>☐ About once every other week (about 12 times)<br>☐ About once every month (about 6 times)<br>☐ Less than once a month (about 3 times)<br>☐ Once or twice<br>☐ Never
8. Number of times you had an appointment with a specialist gynaecology nurse at the hospital	☐ More than once a week (about 50 times)<br>☐ About once a week (about 25 times)<br>☐ About once every other week (about 12 times)<br>☐ About once every month (about 6 times)<br>☐ Less than once a month (about 3 times)<br>☐ Once or twice<br>☐ Never
9. Number of times **you** telephoned a specialist gynaecology nurse at the hospital	☐ More than once a week (about 50 times)<br>☐ About once a week (about 25 times)<br>☐ About once every other week (about 12 times)<br>☐ About once every month (about 6 times)<br>☐ Less than once a month (about 3 times)<br>☐ Once or twice<br>☐ Never
10. Number of times you had contact with any other hospital services (e.g. psychologist, physiotherapy, doctor's secretary)<br><br>Please say who you contacted<br><br>________________________	☐ More than once a week (about 50 times)<br>☐ About once a week (about 25 times)<br>☐ About once every other week (about 12 times)<br>☐ About once every month (about 6 times)<br>☐ Less than once a month (about 3 times)<br>☐ Once or twice<br>☐ Never

11. Do you have any comments that you would like to make?

____

____

____

____

____

____

____

Thank you

Please return the questionnaire in the envelope provided.

If you have any questions about this study please contact:
Kinta Beaver
Professor of Cancer Nursing
School of Health
University of Central Lancashire
Brook Building
Preston. PR1 2HE
Tel: 01772 893715; E-mail: kbeaver@uclan.ac.uk
