# Supplementary material for: Cost-Consequence Analysis Alongside a Randomised Controlled Trial of Hospital Versus Telephone Follow-Up after Treatment for Endometrial Cancer
Source: Appl Health Econ Health Policy. 2018 Apr 12;16(3):415–27. doi: 10.1007/s40258-018-0378-6 (PMC5940716; doi:10.1007/s40258-018-0378-6)
Supplement: Supplementary file 2 — Supplementary material 2 (DOC 74 kb) [file 40258_2018_378_MOESM2_ESM.doc]

**FURTHER INFORMATION ON UNIT COSTS**

This appendix provides further details on elements of the unit costing.

**Doctor costs**

Salary costs for doctor-led consultations are based on the mean basic salaries reported in [1]. They were not adjusted for overtime, shift work or geographic allowances in the basecase. Costs of these consultations have been adjusted using the non-London multiplier. Costs for doctors include the costs of qualifications. [1] does not provide information on the ratio of direct to indirect time for consultants, registrars or F2/F1 doctors. We assumed that hospital and telephone appointments generated the same amount of non-patient contact work (e.g. paperwork). In the absence of consistent estimates between nurses and doctors [1], we assumed that nurses and all grades of doctor spent 55% of their time in direct patient contact [2].

Some respondents indicated that they saw a ‘hospital doctor’, with no information as to seniority. These responses were costed as the simple (i.e. unweighted) average of the costs of consultants, registrars and F2/F1 doctors.

**Nurse costs**

A senior hospital nurse was assumed to be remunerated at Band 7. Band 8 nurses were assumed to be remunerated at band 8a. Specialist nurses in community practice were costed using data on such nurses reported in [1], while specialist nurses in hospital practice, whether the specialism was described specifically or not were costed as Band 7 nurses. Telephone consultations with specialist nurses were costed in the same way, but excluded overheads. Resource use described by patients as hospital incontinence clinics were costed as one hour of patient time with a hospital specialist nurse.

**Costs of other healthcare professionals**

Ratios of direct to indirect time were taken from [1], where available, or the most recently available in other editions of Curtis. Hospital and community psychologists, and psychosexual counsellors, were costed in the same way, using data from [1] on the costs of community psychologists. Durations of consultation, where not provided in the trial were based on the most recently available data in [1-3], and documented assumptions where information was not available from these sources.

**Transport**

Private car rates are based on HMRC mileage and fuel allowances (taken from https://www.gov.uk/government/publications/rates-and-allowances-travel-mileage-and-fuel-allowances/rates-and-allowances-travel-mileage-and-fuel-allowances)

Hospital parking rates are based on a simple average of the costs of parking for two hours at each of the hospital sites involved in the ENDCAT trial. The information was obtained from the websites of each hospital. Hospital consultations are recorded as being of shorter duration than two hours, but some patients indicated that they experienced delays in starting their consultations.

Hospital transport services are based on values reported in [4], which were assumed to relate to a one-way trip, and were inflated to 2016/17 price levels.

**Tests**

Tests described as ‘histology’, high vaginal swabs, abdominal wounds swabs and bile swabs are costed as directly accessed pathology services with currency code DAPS2 (‘histopathology and histology’), ‘bloods’ as directly accessed pathology services currency code DAPS3 (‘integrated blood services’), and mid-stream urine sample as directly accessed pathology services currency code DAPS4 (‘clinical biochemistry’).

Ultrasonography was costed as a weighted average of scans over and under 20 minutes in the urology service description. ‘Biopsy’ was costed as diagnostic hysteroscopy with biopsy.

**Other resources**

Complementary therapy – in the absence of information about exactly what therapies were used – was costed as the mean value of non-consultant led-outpatient attendances in NHS hospitals, based on data in NHS Reference Costs. A resource described as ‘urodynamics’ was costed from Reference Costs as ‘Dynamic Studies of Urinary Tract, 19 years and over’ in the urology service description. A resource described as ‘hospital genetics’ was costed from Reference Costs as a weighted average over all attendances of non-consultant led outpatient attendances in the clinic genetics service description.

The costs of a NHS walk-in centre were taken from [3] and inflated to 2016/17 prices. The cost of mental health services were taken from the weighted average of mental health specialist teams reported in [1]. The costs of hospital pain team use were calculated as the weighted average of complex, major, intermediate and minor pain procedures in the neurology service description from [5].

**FURTHER INFORMATION ON PERSONAL AND OTHER COSTS**

Table A1 provides further detail on NHS and patient travel costs for hospital consultations.

Table A1 NHS and patient travel costs for hospital consultations

| **Mode of transport** | N | Mean return journey costs (£) |
| --- | --- | --- |
| **NHS Transport costs** (% of all NHS transport costs) |  |  |
| Hospital transport | 3 (100%) | 28.85 |
| **Patient travel costs** (% of all personal transport costs) |  |  |
| Car (including hospital parking costs) | 88 (79.3%) | 12.99 |
| Taxi | 4 (3.6%) | 17.44 |
| Bus | 15 (13.5%) | 3.31 |
| Walking | 3 (2.7%) | 0.00 |
| Train | 1 (0.9%) | 10.62 |
| **Weighted average cost of transport to hospital** |  | 11.47 |

Table A2 provides further detail on productivity costs for different types of consultation.

Table A2 Productivity costs for different types of consultation

|  | Telephone consultation | | Hospital consultation | |
| --- | --- | --- | --- | --- |
|  | N | Mean cost per patient or companion reporting lost productivity (£) | N | Mean cost per patient or companion reporting lost productivity (£) |
|  |  |  |  |  |
| Patient productivity | 3 | 28.80 | 19 | 56.51 |
| Companion productivity | N/A | N/A | 8 | 63.25 |

**FURTHER INFORMATION ON SUBGROUP ANALYSIS**

Table A3 present further details on the subgroup analysis.

Table A3 Subgroup analysis on 12-month follow up cost data

|  | **Telephone follow-up** | | **Hospital follow-up** | |  |
| --- | --- | --- | --- | --- | --- |
| **Subgroup** | **n** | **Mean cost per patient** | **n** | **Mean cost per patient** | **Mean difference in follow-up costs between arms (95% bca CI)** |
| **Duration of routine follow-up** | | | | | |
| Less than 6 months | 59 | £93 | 58 | £106 | -£13 (-£77 to £38) |
| 6 months or longer | 37 | £59 | 40 | £37 | £23 (£11 to £42) |
| **Age** | | | | | |
| Age less than 70 | 61 | £85 | 80 | £84 | £1 (-£46 to £50) |
| Age equal to or greater than 70 | 35 | £70 | 18 | £49 | £21 (£9 to £39) |
| **Education** | | | | | |
| No qualifications | 14 | £71 | 15 | £41 | £30 (£13 to £54) |
| Qualifications below degree level | 74 | £81 | 78 | £87 | -£6 (-£55 to £36) |
| Degree holders | 7 | £77 | 5 | £46 | £31 (-£2 to £100) |
| **Employment status** | | | | | |
| Actively working | 30 | £102 | 41 | £67 | £34 (-£10 to £161) |
| Not actively working | 66 | £70 | 57 | £85 | -£15 (-£90 to £17) |
| **Occupational group** | | | | | |
| Group 1 - Managerial/Professional | 37 | £68 | 36 | £94 | -£26 (-£137 to £11) |
| Group 2 - Administration/Skilled/ Trades/Caring/Sales | 47 | £86 | 43 | £7 | £10 (-£55 to £68) |
| Group 3 - Operatives/Elementary occupations | 8 | £68 | 16 | £46 | £22 (£3 to £52) |
|  |  |  |  |  |  |

References

1. Curtis L. Unit Costs of Health and Social Care 2013. Canterbury: Personal Social Services Research Unit; 2013.

2. Curtis L. Unit Costs of Health and Social Care 2010. Canterbury: Personal Social Services Research Unit; 2010.

3. Curtis L. Unit Costs of Health and Social Care 2011. Canterbury: Personal Social Services Research Unit; 2011.

4. Liu F, Treharne C, Culleton B, Crowe L, Arici M. The financial impact of increasing home-based high dose haemodialysis and peritoneal dialysis. BMC Nephrology. 2014 2014/10/02;15(1):1-12.

5. Department of Health. Reference Costs 2012/13. London2013.
